# A comprehensive collection of experimentally validated primers for Polymerase Chain Reaction quantitation of murine transcript abundance

**DOI:** 10.1186/1471-2164-9-633

**Published:** 2008-12-24

**Authors:** Athanasia Spandidos, Xiaowei Wang, Huajun Wang, Stefan Dragnev, Tara Thurber, Brian Seed

**Affiliations:** 1 Center for Computational and Integrative Biology, Massachusetts General Hospital. MA, USA; 2 Department of Genetics, Harvard Medical School, 185 Cambridge Street, Boston, MA 02114-2790, USA; 3 Division of Bioinformatics and Outcomes Research, Department of Radiation Oncology, Washington University School of Medicine, 4921 Parkview Place, St. Louis, MO 63110, USA; 4 Idearc Media Corp, 1601 Trapelo Road, Waltham, MA 02451, USA

## Abstract

**Background:**

Quantitative polymerase chain reaction (QPCR) is a widely applied analytical method for the accurate determination of transcript abundance. Primers for QPCR have been designed on a genomic scale but non-specific amplification of non-target genes has frequently been a problem. Although several online databases have been created for the storage and retrieval of experimentally validated primers, only a few thousand primer pairs are currently present in existing databases and the primers are not designed for use under a common PCR thermal profile.

**Results:**

We previously reported the implementation of an algorithm to predict PCR primers for most known human and mouse genes. We now report the use of that resource to identify 17483 pairs of primers that have been experimentally verified to amplify unique sequences corresponding to distinct murine transcripts. The primer pairs have been validated by gel electrophoresis, DNA sequence analysis and thermal denaturation profile. In addition to the validation studies, we have determined the uniformity of amplification using the primers and the technical reproducibility of the QPCR reaction using the popular and inexpensive SYBR Green I detection method.

**Conclusion:**

We have identified an experimentally validated collection of murine primer pairs for PCR and QPCR which can be used under a common PCR thermal profile, allowing the evaluation of transcript abundance of a large number of genes in parallel. This feature is increasingly attractive for confirming and/or making more precise data trends observed from experiments performed with DNA microarrays.

## Background

Quantitative polymerase chain reaction (QPCR) has become a widely applied technique for quantitative gene expression analysis [1,2]. The technique is frequently used to validate and improve the precision of measurement of differences in transcript abundance detected by DNA microarray experiments [3]. In QPCR, product formation is monitored at the end of each thermal cycle by determining the strength of a fluorescent signal that is proportional to the amount of product [4,5]; QPCR thus provides more information than can be inferred from signal detected at the end of multiple cycles of reaction, as in conventional PCR analysis [6-8]. Because data can be collected from the exponential phase of the reaction a generally reliable quantitation of target DNA concentration can be achieved [9]. Detection of QPCR product concentration is usually accomplished by one of two general fluorescence-based approaches: the measurement of a target sequence-selective signal arising from a conformational change in a labeled primer, or the measurement of total DNA formed during the reaction. In the former method, target-specific probes containing fluorophores, such as hydrolysis probes [10-13], dual hybridization probes [14], molecular beacons [15] or scorpions [16,17] are designed. These detection systems provide partial protection against the risk of generation of signals from off-target amplicons but the primers are considerably more expensive to generate than conventional unlabeled primers. In a more widely practiced variant of QPCR, sequence non-selective fluorescent dyes that bind to double-stranded DNA, such as SYBR Green I, are used [18,19]. The quantum yield of SYBR Green I dye intercalated into double-stranded DNA is much greater than the quantum yield of free dye, leading to an increase in fluorescence intensity that, at saturating dye concentration, is proportional to DNA concentration [20]. This yields a simple inexpensive way to measure product amplicon formation. However, the contribution of fluorescence from DNA arising by amplification of undesired sequences cannot be determined without some additional measure, such as thermal dissociation analysis [21].

Several online resources have been described that can be used to design primers for PCR and QPCR [22-25] and are useful for gene expression analysis, when a small number of genes are of interest. We have previously described a resource of designed primers that can be used for real-time PCR with sequence independent detection methods, such as SYBR Green I detection, and that can work under a common PCR thermal profile [26]. Amplification of undesired sequences is a common problem in QPCR, and poses greater difficulties when the amplification conditions cannot be tailored to the primer pair of interest, as for example would be the case for massively parallel QPCR. The primer design algorithm used for the selection of primers for this study was based on a previous approach to the prediction of oligonucleotides for the study of protein coding regions by microarrays [27], but differed by the addition of filters thought to be important for PCR primer specificity. Primers were designed from cDNA sequence information and the principal filter for cross-reactivity was the rejection of primers containing contiguous residues (15 bases or longer) present in other sequences [27]. Additionally, the selected primer pairs had no self-complementarity, low 3' end stability and high complexity. Low complexity regions may contribute to primer cross-reactivity [28], so they were excluded using the DUST program [29]. The primer *T*_m_s were in the same range, as well as their GC contents. Short amplicons (60–350 bp) were favored during primer selection, but in some cases 100–800 bp amplicons were also considered when the design criteria could not be met for shorter amplicons.

The collection of designed primer pairs has been deposited in a public resource called PrimerBank [26]. PrimerBank  contains primers for most known human and mouse genes (Table 1). The primers designed for the mouse genome cover 27684 genes, but because of some redundancy – one primer pair can represent multiple genes, in most cases isoforms – only 26855 primer pairs were synthesized to represent once each of these 27684 genes (Table 2). For another 1165 mouse genes, it was not possible to design primers, mainly due to low sequence quality. The average sequence length for these genes, the majority of which are 'unknown' or RIKEN sequences, is 435 bp while the average mouse gene has 1293 bp. All primers have been designed to have uniform properties and work using the same PCR conditions which simplifies analyzing the expression of many genes in parallel by QPCR.

**Table 1:** Statistics of primers contained in the PrimerBank database.

**Species**	**Genes represented**	**Primers**
Human	33741	167882
Mouse	27684	138918
Total	61425	306800

**Table 2:** PrimerBank primer design and gene representation.

**Mouse primer pair design**	**Number of mouse primer pairs or genes**
Primer pairs with no redundancy	23700
Primer pairs with 2 target genes	2534
Primer pairs with more than 2 target genes	621
Total number of primer pairs	26855
Total number of genes represented	27684
Total number of genes not represented	1165

Primer pairs for the mouse genome have 27684 gene targets. Design constraints allowed only 26855 primer pairs to be synthesized, some of which amplify the same sequence from 2 genes or gene isoforms.

**Figure 1:**
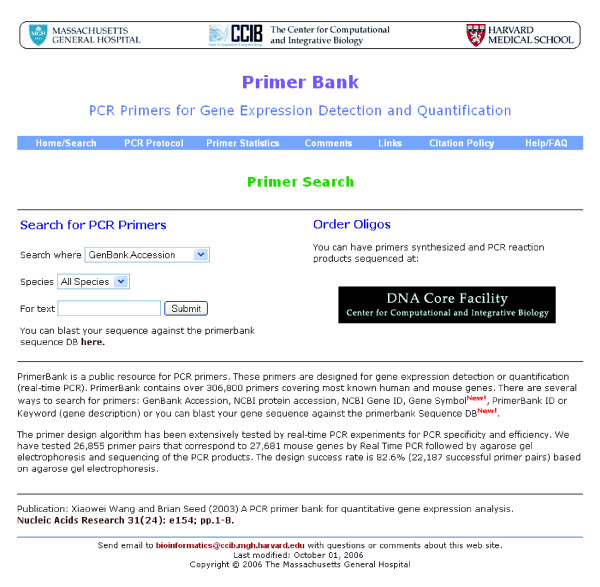
**A screenshot of the web interface for PrimerBank**. Several primer search terms can be used, such as: GenBank accession number, NCBI protein accession number, NCBI gene ID, PrimerBank ID, NCBI gene symbol or gene description (keyword). Website: [26].

Previously we tested by conventional and QPCR 112 primer pairs from PrimerBank representing 108 genes [26]. These primers amplified successfully and specifically the genes for which they had been designed, even though some genes were from closely related gene families. As a second step, we tested by QPCR 26855 PrimerBank mouse primer pairs, representing most known mouse genes, in order to determine if they can successfully amplify the genes for which they had been designed. From the experimental validation procedure, we identified 17483 pairs of primers that amplify unique sequences corresponding to distinct murine transcripts. We also validated on genomic DNA some of the primer pairs that initially failed by QPCR, to provide explanations for these failures. We determined the uniformity of amplification using 96 PrimerBank primer pairs, and the technical reproducibility of the QPCRs, using the same primer pairs. In addition, SYBR Green I sequence specificity was investigated, using a set of sequences differing in length and base composition. Successful primer pair information is now freely available from the PrimerBank database together with the experimental validation data (Figure 1). The mouse serves as an excellent model for studying the function of human genes *in vivo *[30] and currently more genomic resources exist for mouse compared to human. The experimental validation of PrimerBank mouse primers can be applied to functional analysis of human genes.

## Results

### High-throughput primer validation procedure

**Figure 2:**
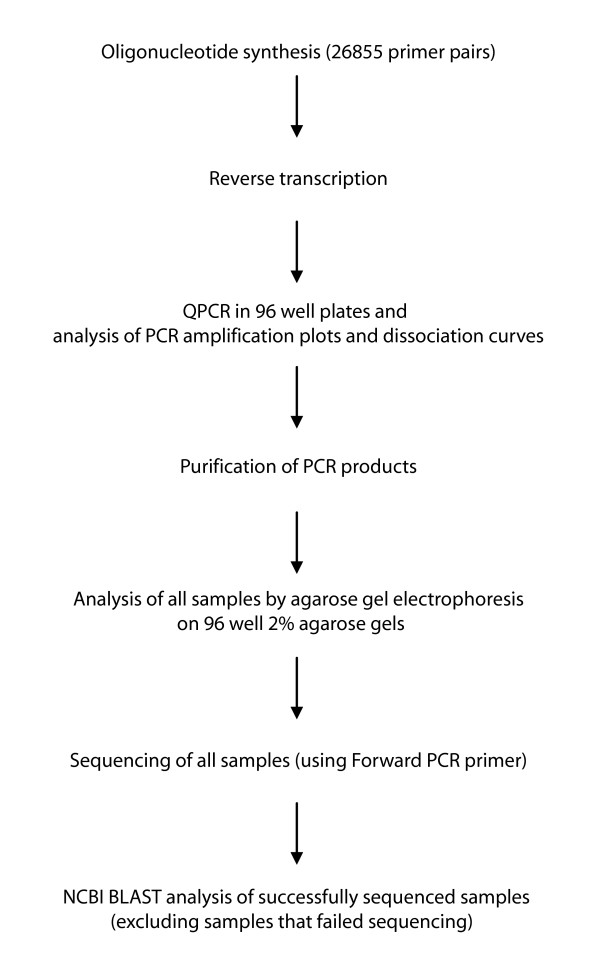
**Summary of procedure for experimental validation of PrimerBank mouse primers**.

A collection of primer pairs from PrimerBank covering most known mouse genes was tested by QPCR, agarose gel electrophoresis, sequencing and BLAST. An overview of the procedure used for primer validation can be seen in Figure 2. Universal mouse total RNA was reverse transcribed using random hexamers and the cDNA was used as a template. 26855 primer pairs, corresponding to 27684 transcripts, were tested by QPCR and the amplification plots and dissociation curves were analyzed. The same PCR conditions were used for all reactions. PCR amplification plots indicate SYBR Green I fluorescence which is proportional to PCR product formation. Dissociation curves indicate the loss of SYBR Green I fluorescence as the PCR product duplex dissociates. *T*_m_ and the shape of the dissociation curve are a function of GC content, sequence and length [2,31]. From the amplification plots, PCR products appeared typically between 19 and 27 cycles of PCR, with a small variation of 1 or 2 cycles depending on the length of the PCR product and thus the amount of SYBR Green I bound to it. As a general observation, most shorter length products (from 60 bp) appeared between 20 and 27 cycles and their *T*_m_s were between 75°C and 85°C, and most longer length products (>200 bp) appeared between 17 and 27 cycles and their *T*_m_s were between 80°C and 90°C.

Agarose gel electrophoresis was used to confirm the correct size of the PCR product, and sequencing and BLAST were used to confirm that the expected transcript had been amplified. All successfully sequenced samples (24476) were BLAST analyzed. From the primer validation procedure, primer pairs were grouped into successful or failed, according to the analysis criteria. From 26855 primer pairs tested 17483 (65.1%) primer pairs, corresponding to 18324 transcripts, were found to be successful by QPCR, agarose gel, sequencing and BLAST analysis. 22189 (82.6%) primer pairs were successful based on agarose gel electrophoresis analysis and 19453 (72.4%) primer pairs were successful based on BLAST analysis. Primer pairs which failed based on the experimental validation procedure can be grouped into various types. Table 3 presents a classification of the types of failures. In a few cases (less than 0.8%), primer pairs were found to be successful based on the gel or BLAST analysis criteria, but no amplification could be detected with SYBR Green I. Sequencing can be very sensitive and a low abundance amplicon can thus be sequenced successfully despite low amounts. Also, in many cases where PCR products were short (~60–80 bp) it was not possible to obtain sequencing information for these samples.

**Table 3:** Classification of failed PrimerBank primer pairs.

Type of failure	Reasons for failure	Number of primer pairs	% from total analyzed
**QPCR failures:**			
QT	No amplification detected	1745	6.5%
			
**Agarose gel failures:**			
G1	No band observed on gel	1619	6.0%
G2	Multiple bands observed on gel	2177	8.1%
G3	Wrong size band observed on gel	645	2.4%
G4	Faint band observed on gel	224	0.8%
GT	Failed based on gel analysis criteria (G1–G4)	4665	17.4%
			
**Sequencing failures:**			
ST	Low sequence quality	2378	8.9%
			
**BLAST failures:**			
B1	Sequences obtained did not match to the expected sequences	1217	4.5%
B2	Low match length between sequences obtained and the expected sequences	2732	10.2%
B3	Low % identity between sequences, expected sequences were not 1^st ^matches	1074	4.0%
BT	Failed based on BLAST analysis criteria (B1–B3)	5023	18.7%

26855 primer pairs, corresponding to 27684 transcripts were tested by QPCR, agarose gel electrophoresis, sequencing and BLAST. 26854 primer pairs, corresponding to 27683 transcripts, were tested by agarose gel electrophoresis, sequencing and BLAST. QPCR failures: QT. Total number of primer pairs for which no amplification was observed using SYBR Green I detection. Agarose gel failures: G1. Primer pairs for which no band could be seen on the agarose gel. G2. Primer pairs for which two or more bands could be seen on the agarose gel. G3. Primer pairs for which one band of the wrong (unexpected) size could be seen. G4. Primer pairs for which a faint band could be seen. GT. Total number of primer pairs which failed based on our gel analysis criteria. Sequencing failures: ST. Total number of primer pairs for which no PCR product sequencing information was obtained (low sequence quality, sequence reads less than 20–30 bases). BLAST failures: B1. Primer pairs whose PCR product sequences obtained did not match to the expected sequence by BLAST. B2. Primer pairs whose PCR product sequences obtained did not match to at least 50% of the length of the expected sequence by BLAST (nearly all for sequence quality reasons). B3. Primer pairs whose PCR product sequences obtained did not match with at least 92% identity to the expected sequence by BLAST, and/or for which BLAST did not return the expected sequence or any known isoforms as the first match. BT. Total number of primer pairs which were not successful based on our BLAST analysis criteria.

A few representative examples of primer pairs are described [see Additional files 1, 2, 3, 4, 5], to demonstrate in detail the analysis of the results generated from the high-throughput primer validation procedure. Data are shown for five successful primer pairs, five primer pairs that failed based on agarose gel electrophoresis analysis and five primer pairs that failed based on BLAST analysis. Information on these primer pairs, such as PrimerBank IDs, primer sequences and amplicon lengths, is shown here [see Additional file 4]. More information on these primers, such as their *T*_m _and location on the gene, can be found in PrimerBank, as well as alternative primer pairs designed for these transcripts.

### PrimerBank user interface

All data generated from the high-throughput primer validation procedure can be freely accessed from PrimerBank . See Figure 1 for the PrimerBank homepage. Users can search the PrimerBank database for primers for their gene of interest using several search terms such as: GenBank accession number, NCBI protein accession number, NCBI gene ID, PrimerBank ID, NCBI gene symbol or gene description (keyword). Search results include primer sequences together with some information about the primers, such as expected amplicon size and *T*_m_. cDNA and amplicon sequences, and validation data can be viewed by clicking on the appropriate links. All validation data can be accessed from PrimerBank, since the validation criteria may be different from the criteria of the users. Also, users can use a BLAST tool found on the PrimerBank homepage (see Figure 1), to find any primers contained in the PrimerBank database that would amplify their sequence of interest. A BLAST tool for the PCR product sequence obtained from the validation procedure can be used to query the NCBI database and this can be found on the validation data webpage. The QPCR and reverse transcription protocols can be found on PrimerBank, as well as a troubleshooting guide.

### Analysis of failed primer pairs

**Figure 3:**
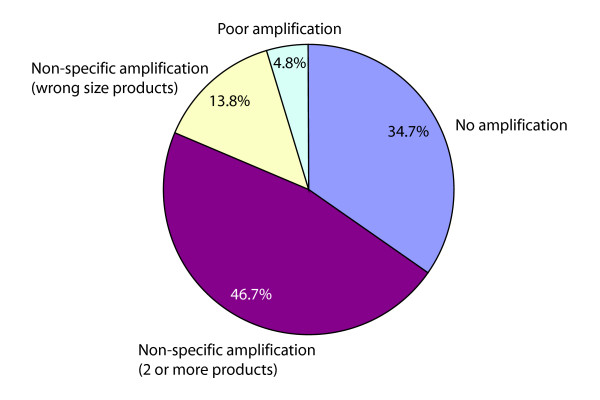
**Distribution of agarose gel failures**. Multiple amplification visualized as two or more bands on the gel accounted for 46.7% of the failed samples. Undesired amplification visualized as the wrong size bands on the gel accounted for 13.8% of the failed samples. Poor amplification visualized as a faint band on the gel was observed in 4.8% of the failed samples and no amplification took place in 34.7% of the failed samples.

A schematic representation of the agarose gel fail distribution can be seen in Figure 3. This analysis was based on determining whether one PCR product of the correct size could be visualized from agarose gel electrophoresis data. Most primer pairs were successful based on at least one step of the primer validation procedure. Two major types of failed primer pairs that comprise most of the failures are primer pairs that failed on agarose gels but were successful by BLAST and primer pairs that failed on BLAST but were successful on agarose gels. 3695 primer pairs failed based on BLAST analysis alone and another 1864 primer pairs failed based on agarose gel analysis alone. In most cases a primer pair failed in one of the analysis steps based on the criteria, but was successful in other analysis steps. The failed samples did not overlap in many cases and this could have been in some cases due to strict BLAST analysis criteria and new splice isoforms seen on the agarose gels. Also, some primer pairs failed by both BLAST and agarose gel analysis, although these are numerically minor. For a detailed description of the analysis criteria see Table 3. The criteria for success or fail may be different from the criteria users might apply and for this reason all validation data can be accessed from PrimerBank.

From the total agarose gel failed reactions, 46.7% were due to multiple amplification products apparent by gel electrophoresis. 13.8% of the total failed reactions were due to undesired amplification, seen as the wrong size band on the gel. 4.8% of the total failed reactions were due to poor amplification, and 34.7% of the total failed reactions were due to no amplification taking place. Multiple or undesired amplifications accounted for the majority (60.5%) of the agarose gel failed reactions. These may represent undocumented transcripts or splice isoforms that could have been amplified in addition to or instead of the expected transcripts. For the reactions that failed because no amplification had taken place, the template sequences may not have been present or present in very low copy number.

### Validation of primer pairs that failed amplification using genomic DNA

From the high-throughput PrimerBank mouse primer pair validation, 1745 samples (6.5%) failed because of no amplification, as seen from the QPCR amplification plots. From the gene description information we found several to belong to olfactory receptors, vomeronasal receptors, transcription factors and low abundance transcripts while others were of unknown function or RIKEN sequences (data not shown). In order to investigate the possibility that the templates for the failed amplification primer pairs were not expressed in the cDNA sample used, we repeated these reactions using genomic DNA as a template. It can be difficult to achieve amplification using genomic DNA as template in general, due to its complexity. However, it can be used successfully if technical difficulties are overcome and can be useful as a universal template as it contains a copy of all genes, and the same amount of template is present for all single-copy genes [32]. We have found that enzymatic digestion (such as *Eco*RI/*Bam*HI digestion used here) can be used for reduction of the complexity of the DNA and thus higher amplification rates. We matched 864 primer pairs to mouse genome sequences obtained from the UCSC genome browser. The remainder of the sequences could not be matched, probably because they were located on exon junctions. 640 of these primer pairs have no *Eco*RI/*Bam*HI restriction sites in their expected PCR amplicons, and were used with *Eco*RI/*Bam*HI digested DNA template to prepare the validation reactions. We tested 192 representative samples, from the 1745 total number of failed primer pair samples, whose expected PCR amplicon lengths range from 60 bp to 123 bp and whose amplicons have no *Eco*RI/*Bam*HI restriction sites. 50 ng *Eco*RI/*Bam*HI digested 129 mouse ES cell genomic DNA was used per 25 μl PCR reaction.

The amplification plots of all 192 samples (2 × 96 well plates) are shown here [see Additional files 6, 7]. The success rate of QPCR based on the amplification plots was high: 88.5% for the first plate [see Additional file 6] and 90.6% for the second plate [see Additional file 7]. However, Ct values differed significantly, from roughly 23 to 40 [see Additional files 6, 7]. The location of the reactions on the plate did not explain this variation. The samples were also analyzed by agarose gel electrophoresis and sequenced (data not shown). Sequences obtained were BLAST analyzed and matched to the expected sequences, confirming that the correct templates had been amplified (data not shown). Therefore, these primer pairs had originally failed because their respective templates were not present in the cDNA sample used and not because of poor primer design, in general.

### Uniformity of amplification and technical replicate tests

We next set out to determine the uniformity of amplification using fully validated PrimerBank primer pairs ie. primer pairs that had been successful in all steps of the validation procedure. 96 primer pairs were chosen with expected PCR amplicon length ranging from 80 bp to 120 bp and containing no *Eco*RI/*Bam*HI restriction sites in their sequences. Both forward and reverse primers were chosen to be on the same exon in order to amplify the same template on genomic DNA. *Eco*RI/*Bam*HI digested 129 mouse ES cell genomic DNA was used as template. After digestion the DNA was purified for PCR by phenol extraction and ethanol/salt precipitation. 50 ng of DNA template was used per 25 μl PCR reaction, which was found by optimization experiments to give a reasonable Ct value.

**Figure 4:**
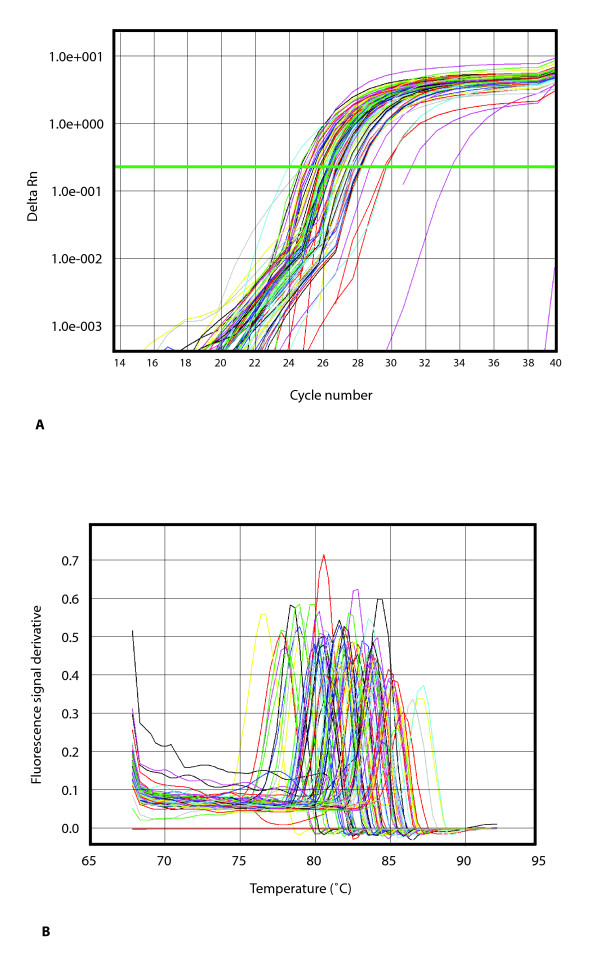
**Uniformity of amplification test using 96 PrimerBank primer pairs**. A. PCR amplification plots. B. Dissociation curves plotted as the raw fluorescence with respect to temperature. Expected PCR product lengths range from 80–120 bp.

**Figure 5:**
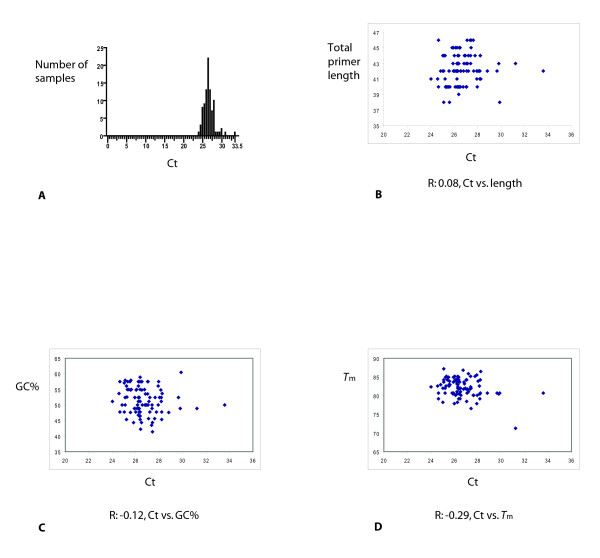
**Analysis of uniformity of amplification test**. A. Ct frequency distribution. B. Correlation of Ct to total primer length, R: 0.08. C. Correlation of Ct to GC%, R: -0.12. D. Correlation of Ct to *T*_m_, R: -0.29.

See Figure 4 for the amplification plots and dissociation curves. As can be seen from Figure 4A, the Ct values for each sample are not exactly the same. This is expected since there will be some stochastic variation. Also, different primer pairs were used for each sample. However, the Ct values are similar, so amplification using PrimerBank primers appears to be relatively uniform. The statistical significance of the difference in Cts observed was determined by plotting a frequency distribution of the number of samples versus the Ct (Figure 5A). A statistical normality test was also used for the analysis of these Ct values, but the data did not pass this test. The effect of primer length and primer GC% on the Ct was studied, by plotting these values against the Ct, and no correlation between these parameters was found (see Figure 5B,C). The effect of the PCR product *T*_m _on the Ct was also studied, by plotting the *T*_m _values against the Ct, and again no correlation was found (see Figure 5D). Since the expected PCR product size varies from 80 bp to 120 bp, some small variation in *T*_m _is expected, and this can be seen from the dissociation curve data (see Figure 4B). The *T*_m _data (obtained from the dissociation curves) was also plotted as a frequency distribution and did not pass the statistical normality test (data not shown).

In order to determine the technical reproducibility of the QPCRs, five 96 well plate assays were prepared using the same technical procedure. Reactions were set up using the same 96 primer pairs and DNA template (129 mouse ES cell *Eco*RI/*Bam*HI digested genomic DNA) that were used for the uniformity of amplification test. The coefficients of variation for each 96 well plate assay are all < 0.1 and the average coefficient of variation for all assays is 0.07 [see Additional file 8]. The individual primer pair Cts for each 96 well plate assay and coefficients of variation are shown here [see Additional file 9]. Ct data from each assay initially did not pass the statistical normality test. The Ct values were normalized, using the formula:

(LnCt - LnCt_av_)/SD,

where LnCt is the natural logarithm of the Ct value used, LnCt_av _is the natural logarithm of the average Ct value of the assay and SD is the standard deviation of the LnCt values for each assay, and outliers were removed. The normalized data passed the normality test, so the data appear to be log normal. The plots of the frequency distributions of the log normal data are shown here [see Additional file 10].

Analysis of pipetting variation during liquid transfer of the fluid handling system was carried out and the transfer efficiency of the robot was found to be 97.3% [see Additional file 11]. The data from the liquid transfer test passed the statistical normality test only after the 9 lowest value outliers were removed (data not shown), but the coefficients of variation are low (less than 0.03) [see Additional file 11]. Variation in liquid transfer can only account for a small amount of the variation observed in QPCR reactions, and hence other factors must be responsible for the differences observed in Ct values.

### SYBR Green I sequence specificity

The SYBR Green I dye has been widely used as a non-sequence specific dye for fluorescence detection of QPCR products [20]. Studies of SYBR Green I-DNA binding showing some sequence specificity of the dye have been reported but these have not been conclusive [20,33,34]. We investigated whether SYBR Green I is sequence specific by adding the dye to a series of amplicons and taking fluorescence readings. 8 amplicons of increasing length and 7 amplicons of increasing AT% [see Additional file 12] were used, whose concentrations were accurately determined (see methods). From these experiments, we did not observe any length dependent or AT/GC dependent sequence specificity of SYBR Green I [see Additional file 13]. However, we cannot exclude the possibility that SYBR Green I can show specificity to sequences such as homopolymer regions of DNA [20] or specific sequences. We also investigated whether SYBR Green I dye binding is sequence specific by estimating the number of PCR product molecules at threshold using the ABI PRISM 7000 Sequence Detection System (Applied Biosystems) [35,36]. For this, the same 14 amplicons as above were used and a template titration series of reactions was prepared for each amplicon. SYBR Green I threshold cycle (Ct) fluorescence will be the same for all amplicons (and all reactions), since the same threshold was used to compare all reactions. However, if SYBR Green I is sequence specific, this fluorescence will correspond to a different number of molecules at threshold for each amplicon. These experiments were inconclusive, as the stochastic error was too large to be able to accurately determine the molecules detected at the threshold (data not shown).

### Estimation of QPCR amplification efficiency

The most common method for the calculation of the amplification efficiency of a QPCR reaction requires preparation of a series of serial dilutions of the sample and creation of a standard curve, whereby efficiency is estimated from the slope of the standard curve [36,37]. However, this method does not provide an accurate value of the efficiency, as the efficiency can vary between different reactions and as input concentration changes. A number of analytical methods have been described for the calculation of the amplification efficiency of a reaction from single reaction kinetics [38] (for a correction in equation 3 of this paper see: [39]), [40-42]. These methods can be more accurate and, when automated, less laborious compared to the standard curve method [43]. Using the following analytical method, we estimated the amplification efficiency values for 13 QPCRs using PrimerBank primer pairs that had been previously used. The log2 fluorescence data was plotted versus the Ct number and the slope of the linear regression was taken to be equal to the efficiency of each reaction [see Additional file 14]. Cycle values closest to the Ct were used, as this region will be the most accurate. The efficiency values ranged from 79% to 96% [see Additional file 14]. Replicates can be used to improve accuracy when using either the standard curve or analytical single reaction kinetics methods [39,44].

We compared amplification efficiency estimation using the standard curve and analytical methods in order to determine the accuracy of each method using the same 13 PrimerBank primer pairs as above [see Additional file 15]. Either the log2 of pg of input template DNA data, for the standard curve method, or the log2 fluorescence data, for the analytical method, was plotted versus the Ct number. Ct was the independent variable and log2 of pg of input template DNA/fluorescence was the dependent variable. The slope of the linear regression was taken to be equal to the efficiency of each reaction. From these results the analytical method shows a smaller variance of efficiency values and the range is smaller compared to the standard curve method [see Additional file 15]. One-way ANOVA analysis was done to determine if amplification efficiency varied significantly between different PrimerBank primer pairs, using each primer pair in a series of titration reactions of template DNA [see Additional file 16]. The average efficiency, standard deviation and coefficient of variation for each group of primer pairs are shown here [see Additional file 17]. The P value is > 0.05 (0.7338) therefore the amplification efficiency is similar between these groups.

In order to account for sample effects, it is useful to provide a model of the experimental measurement of fluorescent PCR product accumulation [45-49]. The following equations can be used:

(1) Log2pgDNA = β_0 _+ β_Ct_x_Ct _+ ε,

where Log2pgDNA is the dependent variable, β_0 _is the intercept, β_Ct _is the regression coefficient for the x independent variable, and ε is the error. Equation 1 can be used for the standard curve method.

(2) Log2Fluorescence = β_0 _+ β_x_x_c _+ ε,

where Log2Fluorescence is the dependent variable, β_0 _is the intercept, β_x _is the regression coefficient for the x independent variable of cycle c, and ε is the error. If β_x _= 1, amplification efficiency is 100%. Equation 2 can be used for the analytical methods.

### PrimerBank primer pair gene location

PrimerBank primer pairs have been designed irrespective of their location on exons. Data from the UCSC genome browser were downloaded and used to find the location of 26854 mouse primer pairs with respect to exons (see Table 4). 19668 primer pairs matched to sequences from the genome browser. Most of the matched primer pairs (16356) are located within exons and at least one primer from the rest of the primer pairs is located on an exon boundary. Primers can be designed to be located on exon boundaries, in order to avoid non-specific amplification of genomic DNA during PCR, but in many cases it was not possible to design primers located on exon boundaries that fulfilled all of the criteria for primer design, most trivially because some transcripts consist of a single exon.

**Table 4:** Primer pair location with respect to exons.

**Primer pairs**	**F primer exon location**	**R primer exon location**
16356	exon	exon
(11235)	(same exon)	(same exon)
1425	exon-exon junction	exon
1576	exon	exon-exon junction
311	exon-exon junction	exon-exon junction

Data from the UCSC genome browser were downloaded and used to find the location of 26854 mouse forward (F) and reverse (R) primer pairs with respect to exons.

## Discussion

### Source of DNA template

A commercial composite mouse RNA preparation was chosen as the source of DNA template for QPCRs, which contains RNA from a panel of eleven different mouse cell types for a good representation of the majority of mouse genes. The composite mouse RNA is composed of total RNA from: whole embryo, embryonic fibroblasts, kidney, liver, lung, B-lymphocyte, T-lymphocyte, mammary gland, muscle, skin and testis. The success rate of the high-throughput PrimerBank primer validation experiments was high as seen both from agarose gel and BLAST analysis. We validated some of the failed reactions using genomic DNA as template, and found that most of the failures in which no PCR product had formed could be due to very little or no cDNA present in the source of DNA template. In order to increase amplification success, specific tissues may be used as sources of cDNA templates where expression of the genes of interest is known.

### Primer specificity

The PrimerBank primer design was based on a successful approach for the prediction of oligonucleotides for the interrogation of protein coding regions by microarrays [27]. However the primer design differs by the addition of filters that are thought to be important for primer specificity [26]. All primers have been designed to work using a relatively high annealing temperature of 60°C and this temperature was used throughout the primer validation experiments described here. High annealing temperatures help reduce non-specific amplification. A high percentage of the total failed samples were due to undesired or multiple amplification, however this may have been for other reasons such as new unidentified genes or splice isoforms. In 3.9% of the cases where multiple bands could be seen on the agarose gel and in 14.6% of the cases where bands of other than the expected size could be seen on the agarose gel, no sequencing information was obtained. Also, 29.7% and 55.2% respectively, did not match to the expected sequences by BLAST. So, sequence homology existed in most cases of undesired or multiple amplification. From the genome-wide primer validation experiments presented here, we have found a high success rate of primer pairs that amplify the transcripts for which they had been designed. For primer pairs that failed because no amplification could be detected, we found that the reason for which they had initially failed was because their target sequences were not present in the target cDNA used. Another reason for failure in the high-throughput validation procedure, may be that protein coding genes in the human genome are fewer than previously thought, and the same may apply to the mouse genome [50].

### A collection of potential new splice isoforms

As mentioned previously, larger than expected or multiple bands were visible on the agarose gel for some samples, however, sequences for these matched confidently by BLAST to the expected sequences. Therefore, the template sequences amplified in these cases could be new genes or splice isoforms. These unrecognized genes or splice isoforms may contribute to primer cross reactivity which results in a lower success rate on the agarose gels. Good primer design depends on accurate genomic information about genes and splice isoforms and it is suggested that many unidentified genes and splice isoforms could exist. All primer pairs that failed because of non-specific amplification, but when BLAST analyzed matched to the expected sequence, could have amplified new non-identified isoforms. This information would be very useful for other researchers, in addition to other strategies for identifying new genes and splice isoforms [51,52]. PrimerBank primers could also be used for determining copy-number variation of a gene or splice isoform [53,54].

### The PrimerBank database

Several online databases exist containing experimentally validated primers, however, only a few thousand primer pairs are currently present in these databases [55-57]. We have previously designed PCR primers for the human and mouse genomes, which are available from PrimerBank [26]. The PrimerBank database currently contains 306800 primers for the mouse and human genomes and is tightly integrated with information from the NCBI databases. PrimerBank has been designed so that researchers can search for primers for their gene of interest using several search terms such as: GenBank accession number, NCBI protein accession number, NCBI gene ID, PrimerBank ID, NCBI gene symbol or gene description (keyword). Currently, all validated primers can be retrieved by searching PrimerBank. In many cases, alternative primer pairs for genes also exist in PrimerBank. NCBI sequences have been attached to the primer information page and NCBI LocusLink indices have been used internally for gene locus mapping. All primers have uniform properties such as *T*_m_, length and GC content and can work using the same PCR conditions.

## Conclusion

We tested by QPCR 26855 PrimerBank mouse primer pairs in order to determine if they can successfully amplify the genes for which they had been designed. We identified 17483 primer pairs that amplify unique sequences that correspond to distinct murine transcripts. All primers have been used under a common PCR thermal profile, allowing the experimentally validated primer collection to be used to evaluate the transcript abundance of a large number of genes in parallel. We used genomic DNA as a template to validate primer pairs that had initially failed by QPCR and provided explanations for the various modes of failure. We determined the uniformity of amplification of the QPCRs using 96 PrimerBank primer pairs. From the uniformity experiments, we found a small variation in Cts which could be due to differences in PCR product length and/or stochastic variation. However, overall amplification appears to be uniform using PrimerBank primers. We investigated the reproducibility of the QPCRs, using the same 96 primer pairs that were used for the uniformity experiments, by comparing Ct values between five technical replicate plates and found coefficients of variation to be low. In addition, SYBR Green I sequence specificity was investigated, using a set of sequences differing in length and base composition. We found no SYBR Green I specificity for the sequences used, but cannot exclude SYBR Green I specificity towards specific sequence motifs. Furthermore, we calculated the efficiency of the reactions from single reaction kinetics data and found the estimated efficiencies to be within a reasonable range, and also that the efficiency can vary between different templates. PrimerBank provides a useful tool for quantitative gene expression analysis by QPCR and facilitates high-throughput studies.

## Methods

### High-throughput primer validation procedure

#### Oligonucleotide synthesis

Oligonucleotides for QPCR were synthesized at Synthesis Core lab of Center for Computational and Integrative Biology at Massachusetts General Hospital. The quality and quantity of the synthesized oligonucleotides were determined by capillary elecrophoresis using the MCE 2000 (CombiSep) instrument and by OD260 reading using the Spectra Max Plus Spectrophotometer (Molecular Devices). Forward and reverse primer mixtures were normalized to 2 μM of each primer for use in QPCR.

#### Preparation of cDNA sample

Universal Mouse Reference total RNA (Stratagene) was used for the preparation of the cDNA sample. Reverse transcription using random hexamers was performed using the Superscript First-Strand Synthesis System for RT-PCR (Invitrogen). Based on the recommended protocol, 20 μg of total RNA was used for each reaction and cDNA samples prepared were in a final volume of 84 μl. The quality of the individual first strand cDNA preparations was tested in a QPCR reaction using mouse actin primers (PrimerBank ID: 6671509a1, 6671509a1F: GGCTGTATTCCCCTCCATCG, 6671509a1R: CCAGTTGGTAACAATGCCATGT).

#### QPCR

QPCRs were performed in polypropylene 96 well plates on the ABI PRISM 7000 Sequence Detection System and ABI 7300 Real-Time PCR System (both from Applied Biosystems). SYBR Green PCR Master mix (Applied Biosystems) or Absolute Q-PCR SYBR Green ROX mix (ABgene) were used. For each reaction, 12.5 μl of the 2× SYBR Green PCR mix were added to 2.5 μl of 2 μM forward and reverse primer mix (final concentration of each primer is 200 nM), 1 μl of cDNA and made to 25 μl with water. The Biomek FX Laboratory Automation Workstation (Beckman Coulter), as well as manual pipetting, was used to prepare the reactions. PCR conditions used were the following: 50°C for 2 minutes (step 1), 95°C for 10 minutes (for Applied Biosystems PCR mix) or for 15 minutes (for ABgene PCR mix) (step 2), 95°C for 15 seconds, 60°C for 30 seconds, 72°C for 30 seconds (step 3 – repeated another 39 times ie. 40 cycles in total). In some QPCRs an additional elongation step was added at 72°C for 10 minutes (step 4). Dissociation curves were obtained by heating and cooling the samples at: 95°C for 15 seconds, 60°C for 30 seconds, 95°C for 15 seconds. DNA was renatured for agarose gel electrophoresis using the following conditions: 50°C for 2 minutes (step 1), 95°C for 15 seconds, 60°C for 30 seconds (step 2 – repeated one more time) and 72°C for 5 minutes (ABI PRISM 7000 Sequence Detection System) or 95°C for 30 seconds, 60°C for 2 minutes (ABI 7300 Real-Time PCR System).

#### Preparation of samples for agarose gel electrophoresis and sequencing

PCR products were purified using Standard Performa 96 well plates and QuickStep 2 SOPE resin (both from EDGE BioSystems), following the recommended procedure.

#### Agarose gel electrophoresis of purified QPCR products

For each sample 10 μl of 2× Orange G loading buffer (composition shown below) was added to 5 μl of the purified PCR product and made to 20 μl with water. Samples were prepared in 96 well plates using the Biomek FX Laboratory Automation Workstation (Beckman Coulter) and using the same instrument applied to 2% agarose 96 well E-gels (Invitrogen). For 10× Orange G loading buffer, a solution of 30% Ficoll 400 (AlfaAesar), 10 mM EDTA (Sigma) was prepared and Orange G dye (Fisher Scientific) was added for color. E-Gel Low Range Quantitative DNA Ladder (Invitrogen) was used as a marker for PCR product size. The gels were run for 12 minutes on the E-Gel 96 Base (Invitrogen) and analyzed using the E-Editor Software (Invitrogen).

#### Sequencing of purified QPCR products

Purified QPCR products were sequenced at Sequencing Core lab of Center for Computational and Integrative Biology at Massachusetts General Hospital.

#### NCBI BLAST analysis

Sequences obtained were BLAST analyzed as batch sets against the NCBI database [58]. In order to identify successful samples, the main parameters considered were the alignment length, the expected sequence match position to the sequence returned by NCBI BLASTn and the percent identity of the two sequences. If more than 50% of the length of the expected PCR product sequence aligned with the expected sequence as first match and there was more than 92% identity between the sequences, this was considered to be a successful sample. In cases where a primer pair had been designed to also amplify a redundant gene and the redundant gene matched first to the sample, the reaction was still considered successful. In these cases the primers have been designed to amplify the same region of the two sequences, so it is not possible to determine by agarose gel or BLAST analysis if one or the other species was amplified during PCR.

### Preparation of digested genomic DNA for QPCR

129 Embryonic Stem cell mouse genomic DNA (isolated by ethanol precipitation) was used. The DNA was digested completely using *Eco*RI and *Bam*HI restriction enzymes. Digests were made by adding 20 μl *Eco*RI buffer (10×) (New England Biolabs), 20 μl 10× BSA, 4 μg DNA, 40 U *Bam*HI (New England Biolabs), 40 U *Eco*RI (New England Biolabs) and water to 200 μl total volume. Digests were incubated at 37°C for 4 hours and 30 minutes and heat inactivated at 75°C for 10 minutes. The digested DNA was phenol extracted and ethanol/salt precipitated. DNA pellets were resuspended in TE pH 8.0.

### QPCRs for uniformity, technical replicate and primer validation tests

QPCRs were performed in polypropylene 96 well plates on the ABI 7300 Real-Time PCR System (Applied Biosystems). For each reaction, 12.5 μl of Absolute Q-PCR SYBR Green ROX mix (ABgene) were added to 2.5 μl of 2 μM forward and reverse primer mix (final concentration of each primer is 200 nM), 1 μl of 50 ng/μl *Bam*HI/*Eco*RI digested genomic DNA and made to 25 μl with water. The following PCR conditions were used: 50°C for 2 minutes (step 1), 95°C for 15 minutes (step 2), 95°C for 15 seconds, 60°C for 30 seconds, 72°C for 30 seconds (step 3 – repeated another 39 times ie. 40 cycles in total), 72°C for 10 minutes (step 4). Dissociation curves were obtained by heating and cooling the samples at: 95°C for 15 seconds, 60°C for 30 seconds, 95°C for 15 seconds.

### Large-scale amplicon preparation for SYBR Green I sequence specificity experiments

Amplicons were prepared large-scale by PCR, in two steps. For the first step PCR, 75 μl PCR reactions were prepared for each sample. For each reaction, 37.5 μl Absolute Q-PCR SYBR Green ROX mix (ABgene) were added to 3 μl of 5 μM primer pair mix (final concentration of each primer is 200 nM), 3 μl universal mouse cDNA (see: 'preparation of cDNA sample' section in methods) and made up to 75 μl with water. The following PCR conditions were used: 95°C for 15 minutes (step1), 95°C for 15 seconds, 60°C for 30 seconds, 72°C for 30 seconds (step 2 – repeated another 39 times ie. 40 cycles in total), 72°C for 10 minutes (step 3). The PCR products were purified using the MinElute PCR purification kit (Qiagen). Purified amplicons were used as templates in large-scale 40× 100 μl PCRs, each reaction containing 50 μl 2× LC1v3 buffer (40 mM Tris-HCl pH8.8, 40 mM KCl, 40 mM ammonium sulfate, 4 mM MgCl_2_, 200 μg/ml BSA, 0.2% Triton X-100, 400 μM dNTP mix, 2.5 M betaine), 4 μl of 5 μM forward and reverse primer mix, DNA template, 1 μl Taq polymerase and water to 100 μl. The PCR conditions used were the following: 95°C for 3 minutes (step 1), 95°C for 15 seconds, 60°C for 30 seconds, 72°C for 30 seconds (step 2 – repeated another 39 times ie. 40 cycles in total), 72°C for 10 minutes (step 3).

PCR reactions were phenol extracted and isopropanol precipitated. DNA pellets were resuspended in TE pH8.0. DNA was purified using Performa DTR Gel Filtration Cartridges (EDGE BioSystems), following the recommended procedure. Amplicon concentrations were determined by taking OD260 readings of each preparation using the ND-1000 Spectrophotometer (Nanodrop). The average value was taken and the OD260 reading from a no DNA template control was subtracted, in order to remove the contribution from primers and buffer components to the spectrophotometric absorption.

### SYBR Green I sequence specificity experiments

DNA samples in 1× Absolute Q-PCR SYBR Green ROX mix (ABgene) were pipetted into OptiPlate-96F black 96 well plates (Perkin Elmer). SYBR Green I fluorescence was detected using the Analyst AD fluorescence plate reader (Molecular Devices) by excitation at 485 nm and emission at 530 nm (505 nm dichroic mirror).

### Robotic and manual liquid transfer test

5 μl of 10 mM dNTP solution were added to 95 μl water and the OD260 readings were taken using the Spectra Max Plus Spectrophotometer (Molecular Devices).

### Primer genome location analysis

Mouse genome sequences were downloaded from the UCSC genome browser [59] and the primer pair sequences were matched by BLASTn to the genome sequences, to identify the primer locations with respect to exons.

## Authors' contributions

AS designed and performed the experiments, analyzed experimental data and prepared the manuscript. XW designed the primer algorithm. HW and SD provided bioinformatics support. TT performed the automation experiments. BS designed and directed the experiments and prepared the manuscript. All authors have read and approved the final manuscript.

## Supplementary Material

Additional file 1 **Five representative examples of primer pairs that were successful throughout the validation procedure.** Click here for file

Additional file 2 **Five representative examples of primer pairs that failed based on agarose gel analysis.** Click here for file

Additional file 3 **Five representative examples of primer pairs that failed based on BLAST analysis.** Click here for file

Additional file 4 **Information for mouse primer pairs from PrimerBank tested using QPCR.** Click here for file

Additional file 5 **NCBI BLAST analysis of successfully sequenced PCR products.** Click here for file

Additional file 6 **Validation of 96 PrimerBank primer pairs which had failed QPCR during the high-throughput validation procedure.** Click here for file

Additional file 7 **Validation of 96 PrimerBank primer pairs which had failed QPCR during the high-throughput validation procedure.** Click here for file

Additional file 8 **Analysis of technical replicate experiments.** Click here for file

Additional file 9 **Analysis of individual primer pairs from technical replicate experiments.** Click here for file

Additional file 10 **Frequency distributions of log normal data from five technical replicate tests.** Click here for file

Additional file 11 **Comparison of pipetting variation between manual and robotic liquid transfer.** Click here for file

Additional file 12 **Amplicons used for SYBR Green I sequence specificity experiments.** Click here for file

Additional file 13 **SYBR Green I binding to dsDNA of increasing length and AT%.** Click here for file

Additional file 14 **Amplification efficiency estimation from single reaction kinetics data.** Click here for file

Additional file 15 **Amplification efficiency estimation using analytical and standard curve methods.** Click here for file

Additional file 16 **One-way ANOVA test to determine if amplification efficiency varies significantly between different PrimerBank primer pairs.** Click here for file

Additional file 17 **PrimerBank primer pair groups used for one-way ANOVA analysis.** Click here for file
