# Supplementary figures and images for: A comprehensive collection of experimentally validated primers for Polymerase Chain Reaction quantitation of murine transcript abundance
Source: BMC Genomics. 2008 Dec 24;9:633. doi: 10.1186/1471-2164-9-633 (PMC2631021; doi:10.1186/1471-2164-9-633)

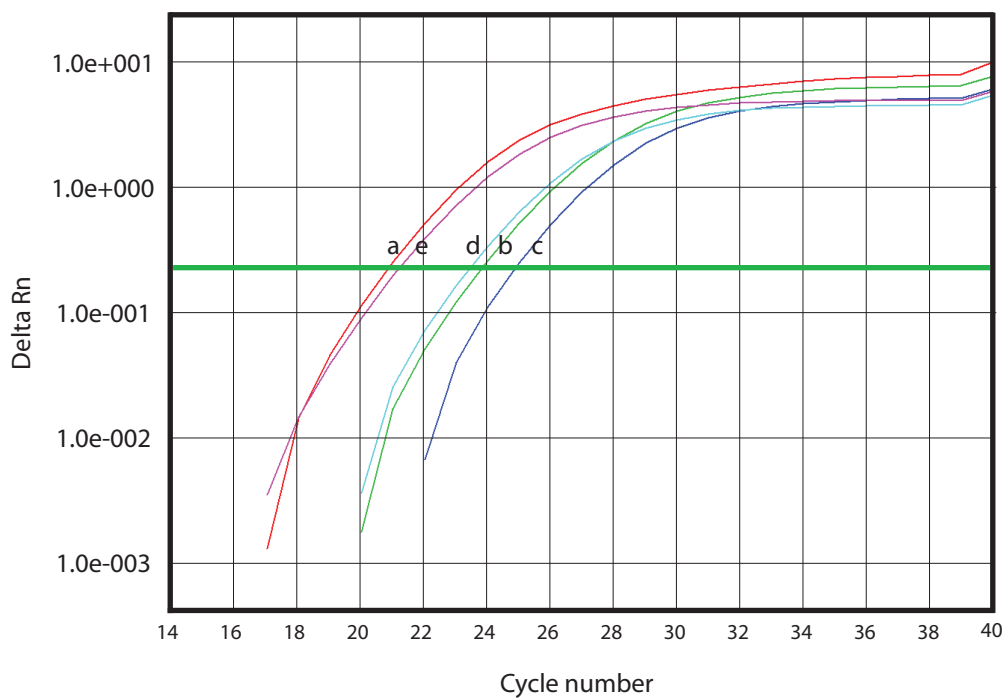

**A**

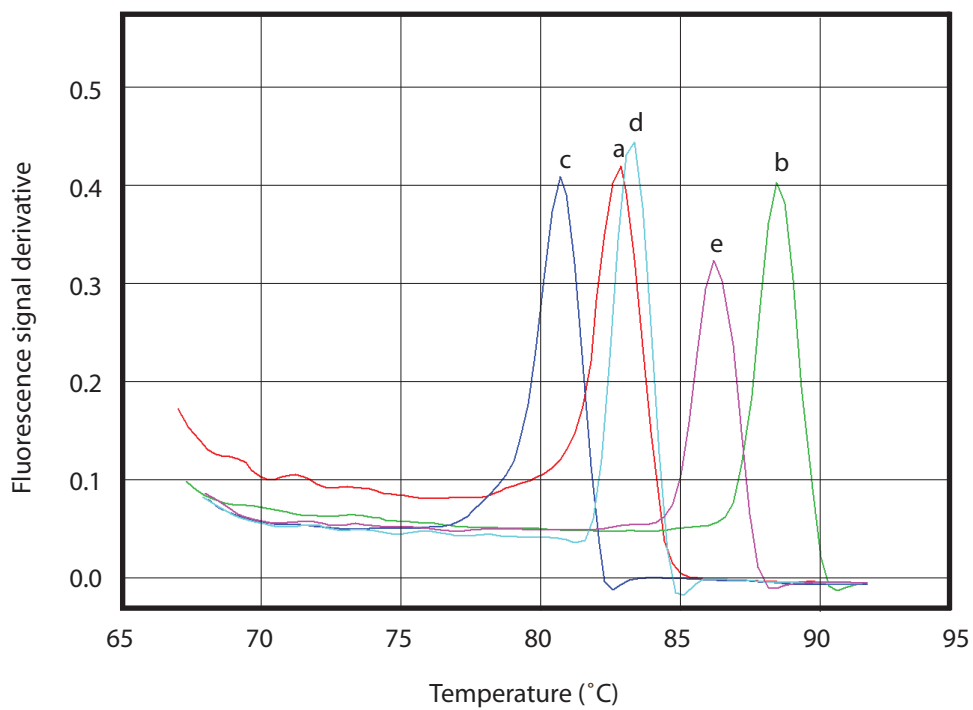

**B**

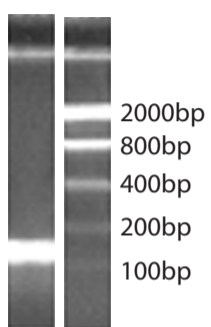

a.

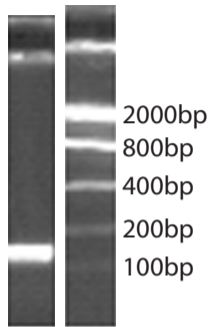

b.

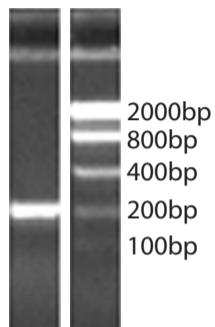

c.

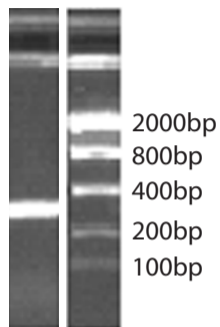

d.

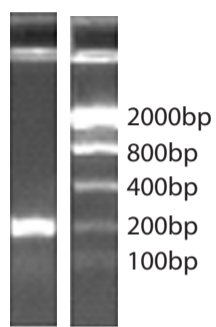

e.

c

Supplement: Additional file 1 — Five representative examples of primer pairs that were successful throughout the validation procedure. [file 1471-2164-9-633-S1.pdf]

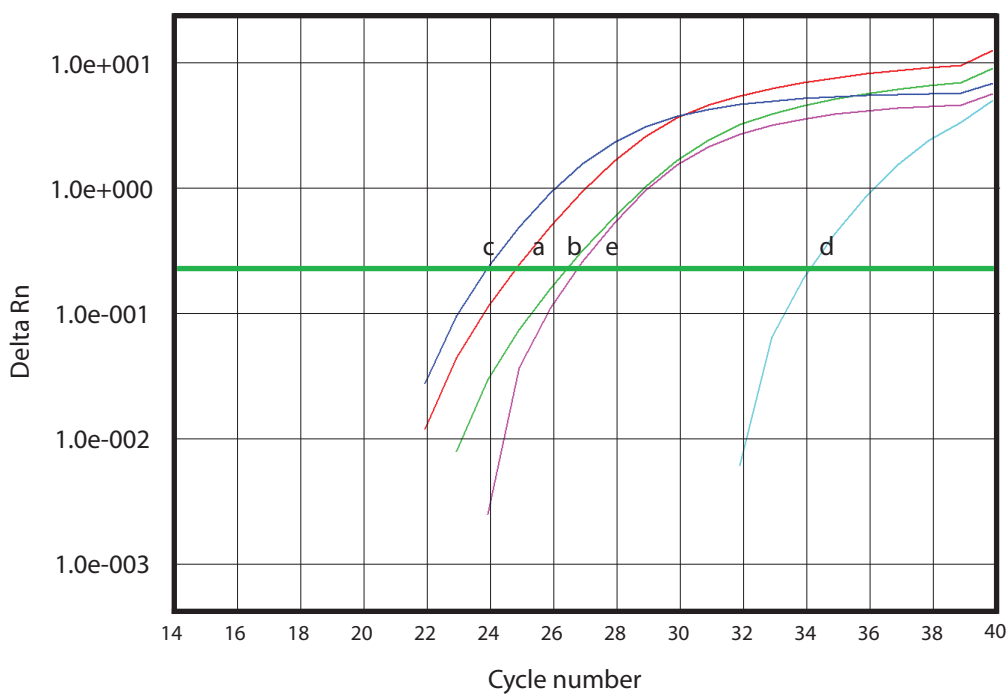

**A**

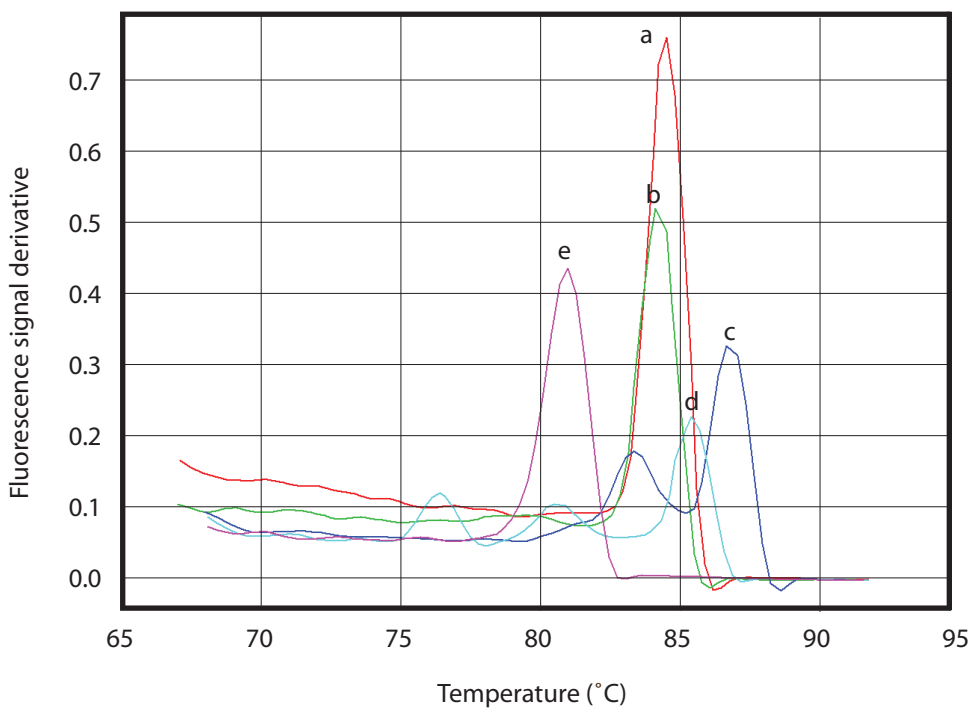

**B**

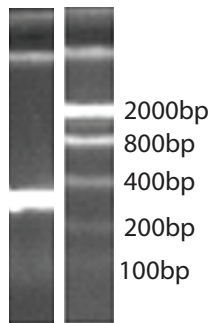

a.

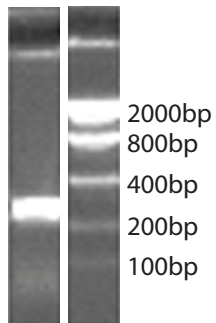

b.

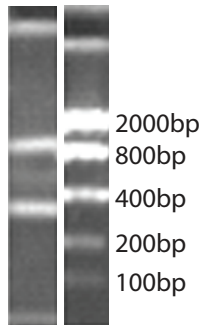

c.

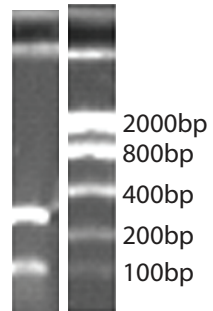

d.

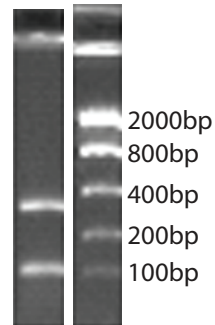

e.

**C**

Supplement: Additional file 2 — Five representative examples of primer pairs that failed based on agarose gel analysis. [file 1471-2164-9-633-S2.pdf]

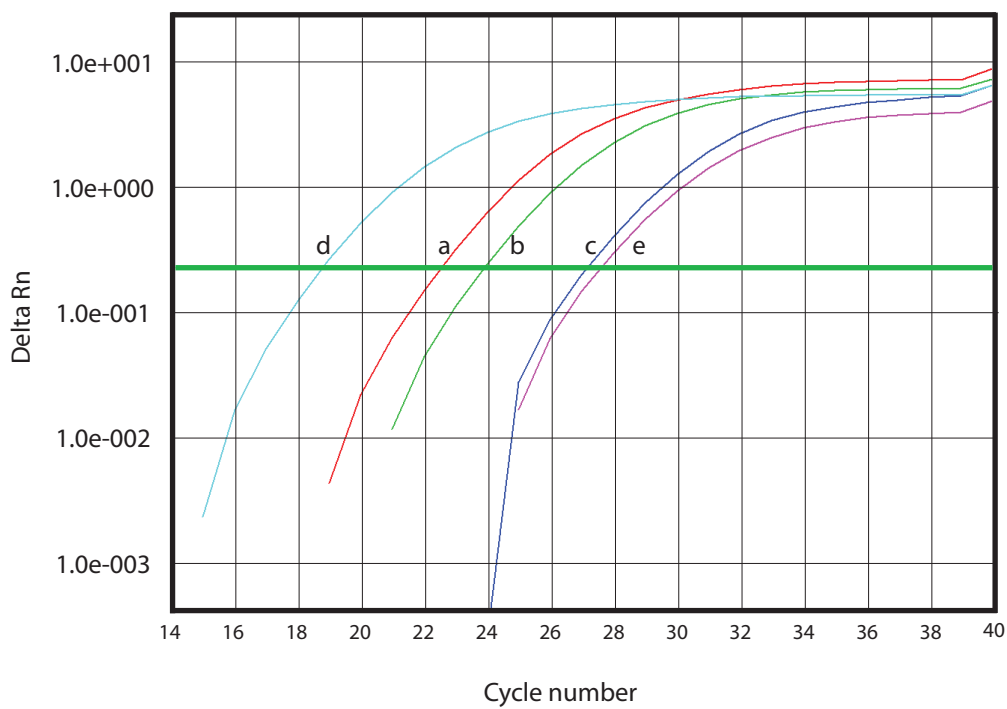

**A**

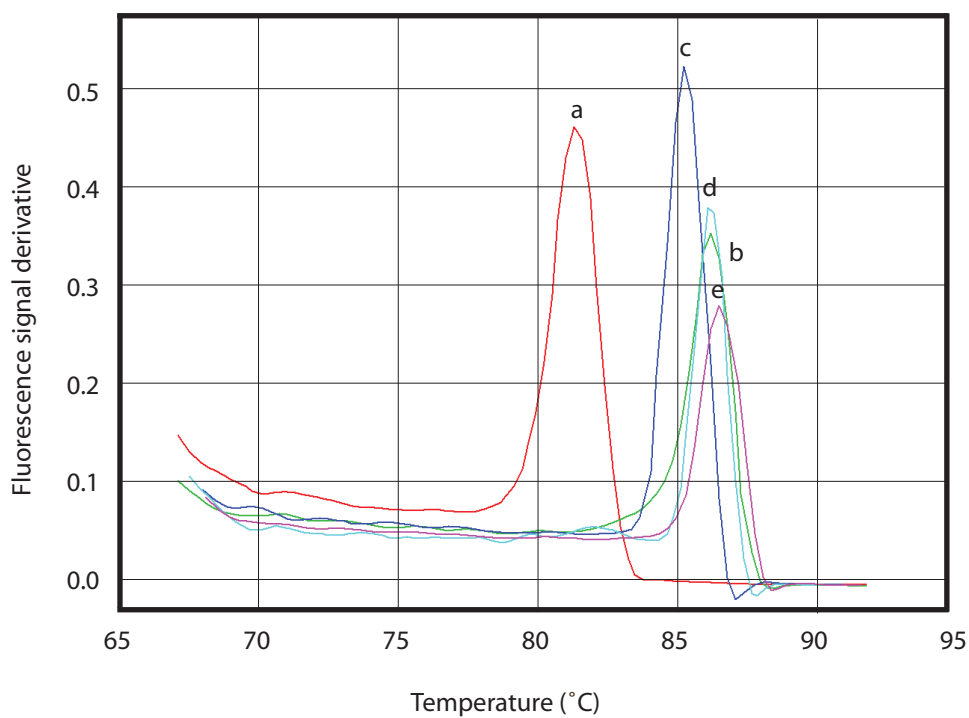

**B**

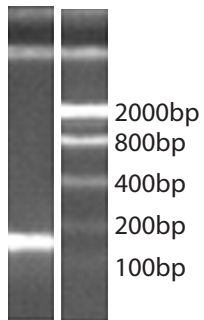

a.

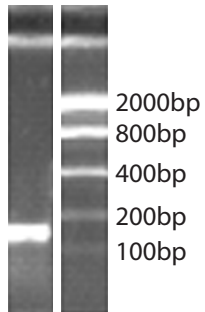

b.

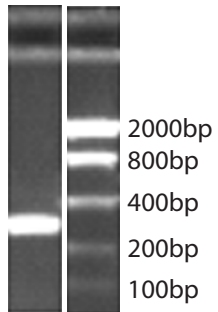

c.

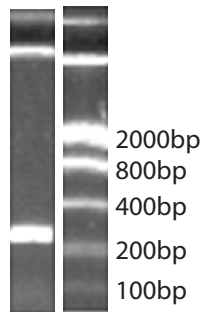

d.

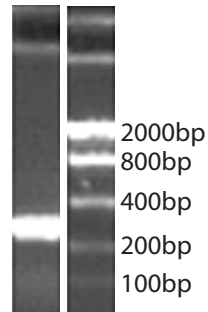

e.

c

Supplement: Additional file 3 — Five representative examples of primer pairs that failed based on BLAST analysis. [file 1471-2164-9-633-S3.pdf]

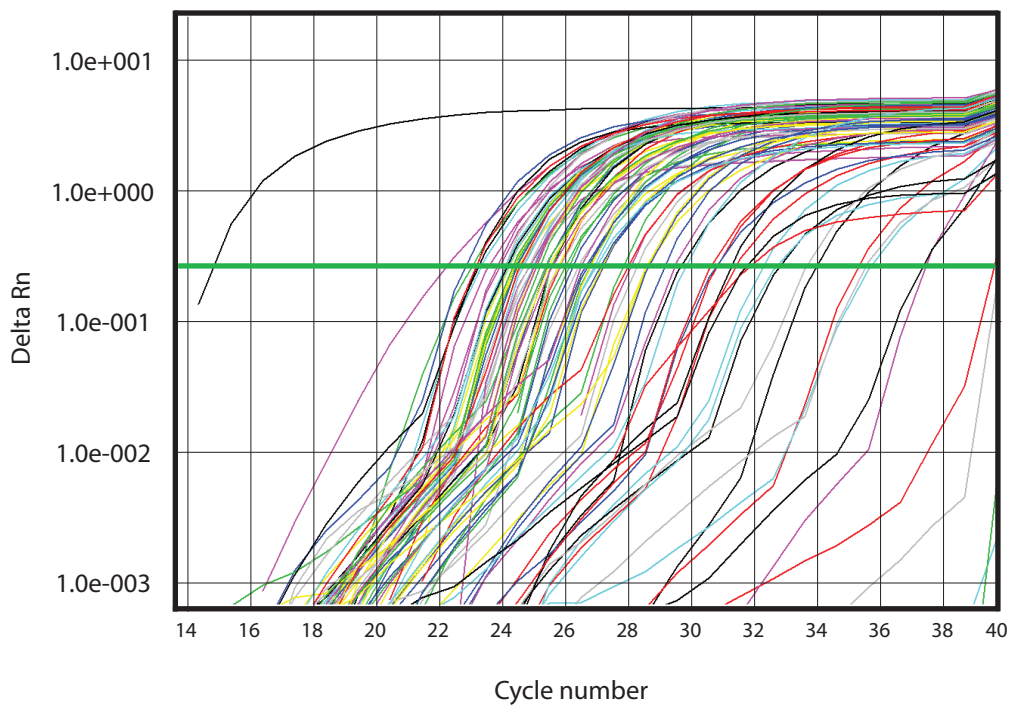

**A**

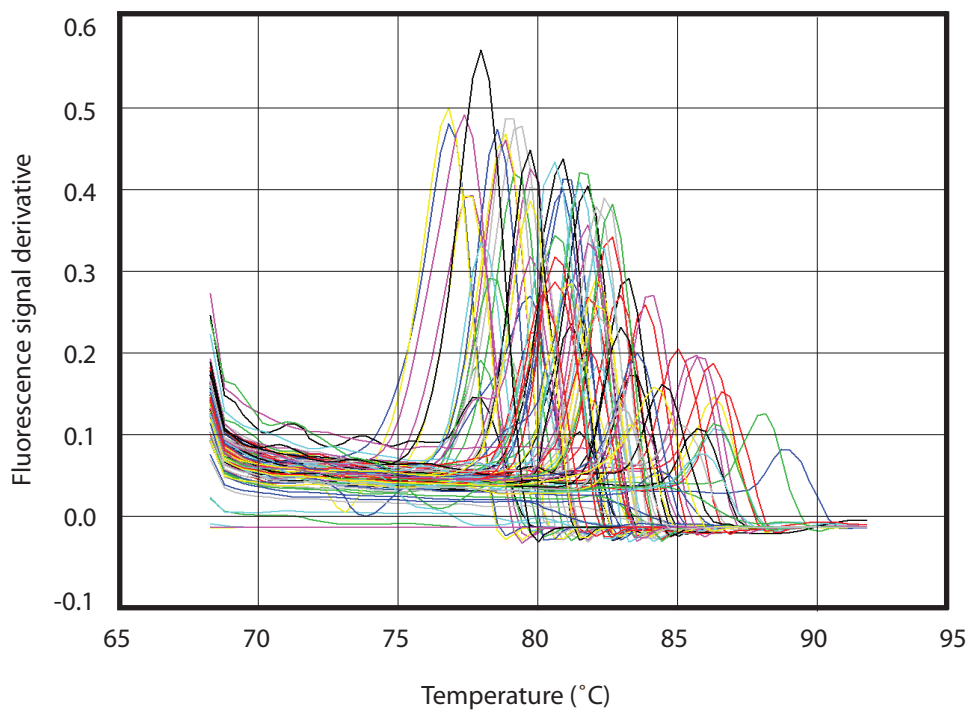

**B**

Supplement: Additional file 6 — Validation of 96 PrimerBank primer pairs which had failed QPCR during the high-throughput validation procedure. [file 1471-2164-9-633-S6.pdf]

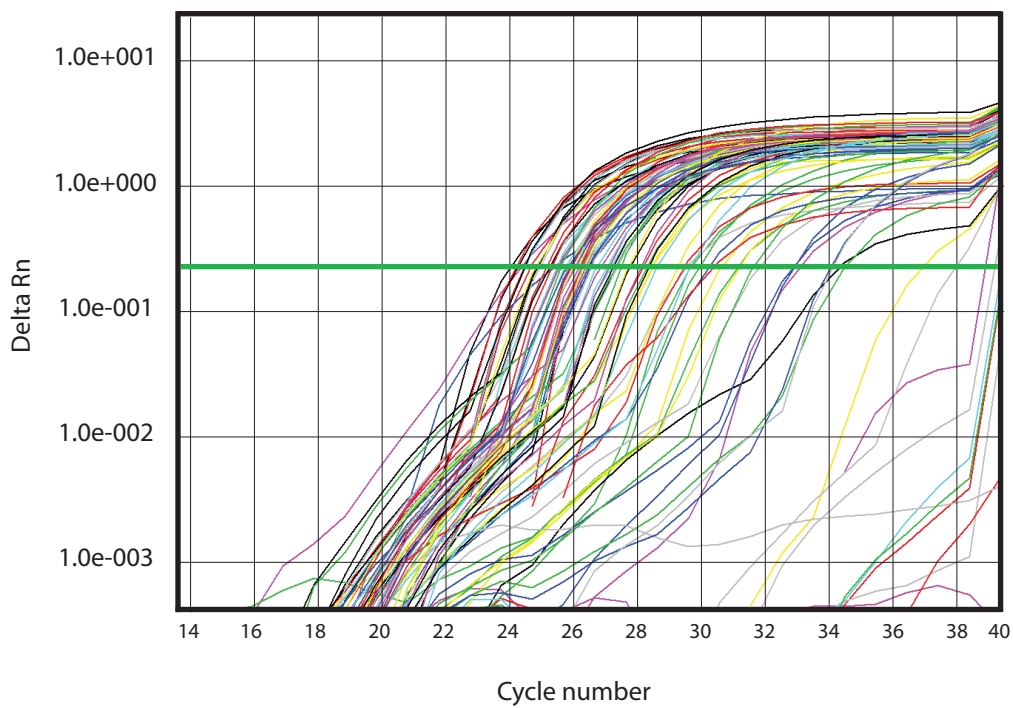

**A**

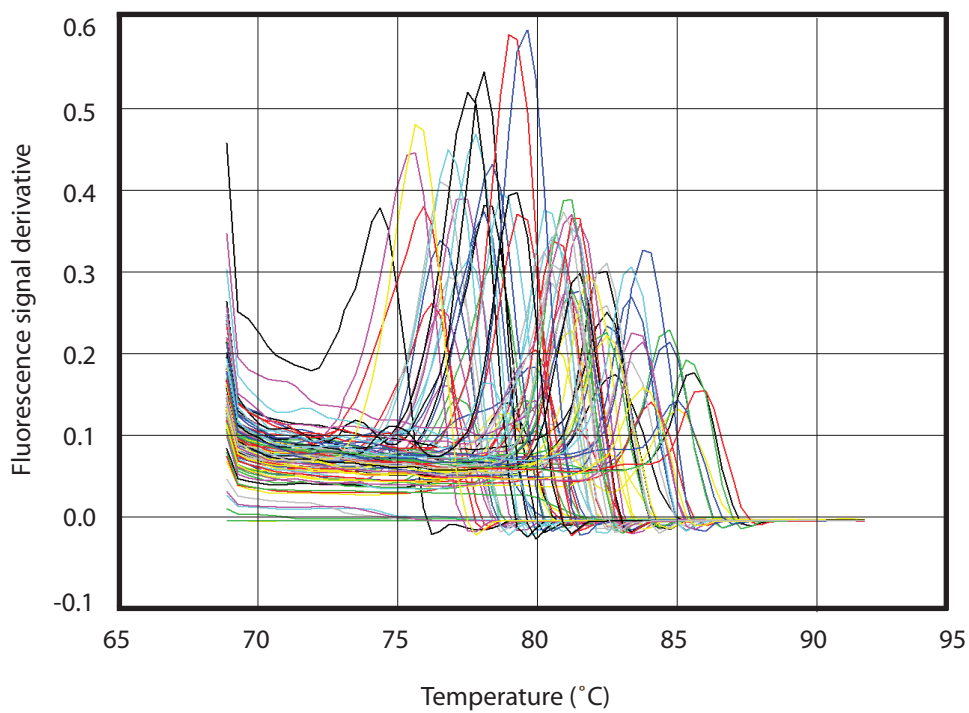

**B**

Supplement: Additional file 7 — Validation of 96 PrimerBank primer pairs which had failed QPCR during the high-throughput validation procedure. [file 1471-2164-9-633-S7.pdf]

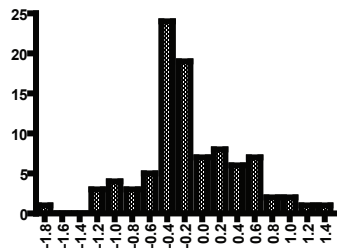

**A**

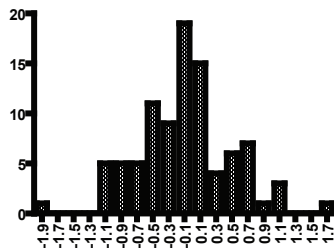

**B**

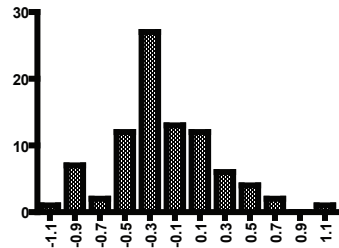

**C**

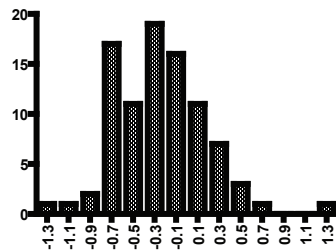

**D**

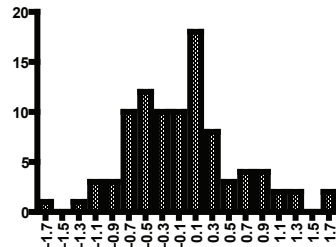

**E**

Supplement: Additional file 10 — Frequency distributions of log normal data from five technical replicate tests. [file 1471-2164-9-633-S10.pdf]

Relative fluorescence units

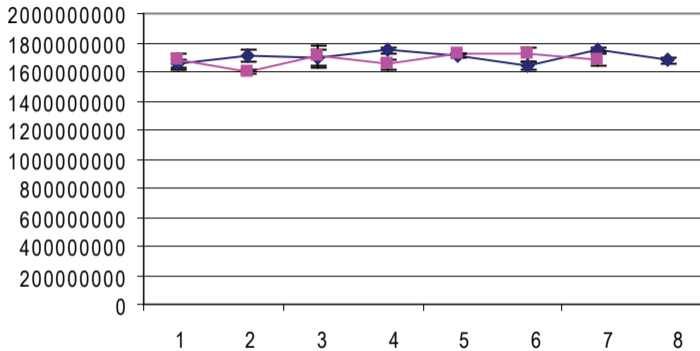

Amplicons

Supplement: Additional file 13 — SYBR Green I binding to dsDNA of increasing length and AT%. [file 1471-2164-9-633-S13.pdf]

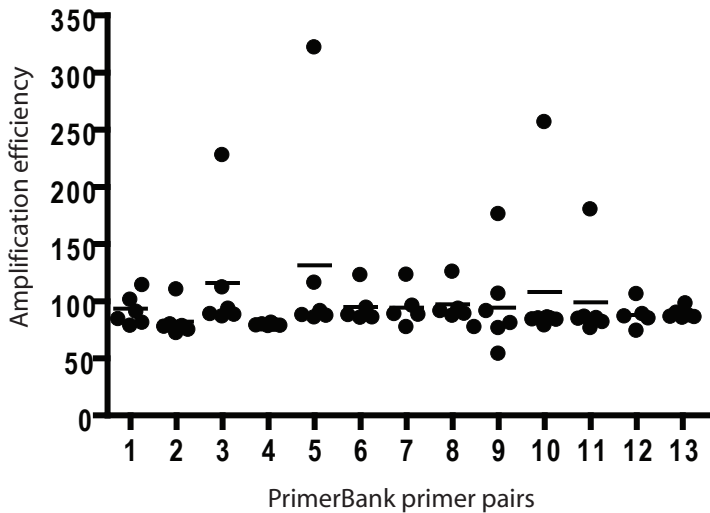

Supplement: Additional file 16 — One-way ANOVA test to determine if amplification efficiency varies significantly between different PrimerBank primer pairs. [file 1471-2164-9-633-S16.pdf]
