# Supplementary material for: A comprehensive collection of experimentally validated primers for Polymerase Chain Reaction quantitation of murine transcript abundance
Source: BMC Genomics. 2008 Dec 24;9:633. doi: 10.1186/1471-2164-9-633 (PMC2631021; doi:10.1186/1471-2164-9-633)
Supplement: Additional file 4 — Information for mouse primer pairs from PrimerBank tested using QPCR. [file 1471-2164-9-633-S4.pdf]

Examples	PrimerBank ID	Protein accession no.	Name	Forward/Reverse primer sequences (5’ to 3’)	Amplicon length (bp)
**Examples of 5 successful primer pairs (Additional file 1):**					
a	6679201a1	NP_032802	platelet-activating factor	AACCCACTTCGAGAGAAAAACC/CCATCAGAATGCACAAACCCA	113
b	30425300a1	NP_780738	RIKEN cDNA F630035L11 gene	GACCCTGCTGACTTTTCGGT/AAGTAGCGTATGCGGTGCTG	143
c	22129479a1	NP_666795	olfactory receptor MOR196-4	CACAGTGACTGGTAACGTGAG/TGGAATTGAATGATGCAGCCA	195
d	26352704a1	BAC39982	unnamed protein product	TCGGGCTCATCGAAGGGTAT/GGTGTGTGGGGTGTGTGATT	295
e	6755010a1	NP_035187	platelet derived growth factor	AAGTGTGAGACAATAGTGACCCC/CATGGGTGTGCTTAAACTTTCG	157
**Examples of 5 failed primer pairs based on agarose gel analysis (Additional file 2):**					
a	28972373a1	BAC65640	mKIAA0734 protein	AGGAACCTGAGGTCACAAACT/GTCTGCCATCCCCCTTTTTCA	114
b	23346543a1	NP_694692	granzyme N	TCCTCTGGATCACACCAATGA/CCCCTGGATTCACCTTGTCTTT	116
c	12832882a1	BAB22297	unnamed protein product	TGGCGTTGGCACAACCTAC/TCACCGGGTTCTCCCTTACC	253
d	53389a1	CAA45973	natural killer cell receptor-P1	CAGCAAGGGTCTACTTTGGTTT/CTTAGCTGGACTGTCTGTTGTTT	266
e	12837565a1	BAB23866	unnamed protein product	CAGAGCAGCTACTAAGCGACT/AAAAGGGGAGATTCGGACAGA	90
**Examples of 5 failed primer pairs based on BLAST analysis (Additional file 3):**					
a	26326251a1	BAC26869	unnamed protein product	GCCATGTGGTGTCATTTTCCT/GTTGTCCAGTCTTGTCCTGAG	114
b	30424726a1	NP_780331	RIKEN cDNA 6330580M05	ACCAGTTCCGGCTCATTGTC/GGCTCGATCTCCACCAAACG	142
c	32766270a1	AAH54855	Unknown	CACTGGCCGTGCTAAACAC/CAGGGGCTAATCGCTCTGG	273
d	12852129a1	BAB29285	unnamed protein product	ATGGGCGGCAACTCTTTGAT/CGTAGCCGGGCTGATTCAT	208
e	15149484a1	NP_150289	protein phosphatase 1	GACAGCTCTTTCGGGCTCAC/CTTCAGATTGCGGTTGGAACT	143
