# Supplementary material for: A comprehensive collection of experimentally validated primers for Polymerase Chain Reaction quantitation of murine transcript abundance
Source: BMC Genomics. 2008 Dec 24;9:633. doi: 10.1186/1471-2164-9-633 (PMC2631021; doi:10.1186/1471-2164-9-633)
Supplement: Additional file 5 — NCBI BLAST analysis of successfully sequenced PCR products. [file 1471-2164-9-633-S5.pdf]

Example	PrimerBank ID	Matched gene (gi number)	% Identity by BLAST	Alignment length	Hit location in matches
**Examples of 5 successful primer pairs (Additional file 1):**					
a	6679201a1	gi|6679201|NP_032802.1	95.6	91	1 of 82
b	30425300a1	gi|30425300|NP_780738.1	98.96	96	1 of 114
c	22129479a1	gi|22129479|NP_666795.1	99.42	171	1 of 253
d	26352704a1	gi|26352704|BAC39982.1	99.62	261	1 of 256
e	6755010a1	gi|6755010|NP_035187.1	99.01	101	1 of 264
**Examples of 5 failed primer pairs based on agarose gel analysis (Additional file 2):**					
a	28972373a1	gi|28972373|BAC65640.1	98.59	71	1 of 254
b	23346543a1	gi|23346543|NP_694692.1	98.81	84	1 of 259
c	12832882a1	gi|12832882|BAB22297.1	92.39	197	1 of 178
c	12832882a1	gi|12832882|BAB22297.1	97.65	85	2 of 178
c	12832882a1	gi|12832882|BAB22297.1	91.35	104	3 of 178
d	53389a1	gi|53389|CAA45973.1	100	175	1 of 269
e	12837565a1	gi|12837565|BAB23866.1	100	48	1 of 258
**Examples of 5 failed primer pairs based on BLAST analysis (Additional file 3):**					
a	26326251a1	No match			
b	30424726a1	No match			
c	32766270a1	gi|32766270|AAH54855.1	99.1	221	2 of 265
d	12852129a1	gi|12852129|BAB29285.1	98.15	162	2 of 266
e	15149484a1	gi|15149484|NP_150289.1	87.76	98	1 of 170
