# Supplementary material for: A comprehensive collection of experimentally validated primers for Polymerase Chain Reaction quantitation of murine transcript abundance
Source: BMC Genomics. 2008 Dec 24;9:633. doi: 10.1186/1471-2164-9-633 (PMC2631021; doi:10.1186/1471-2164-9-633)
Supplement: Additional file 8 — Analysis of technical replicate experiments. [file 1471-2164-9-633-S8.pdf]

96 well plate PCRs	Standard deviation	Average Ct	Coefficient of variation
Technical replicate 1	1.25	22.61	0.055
Technical replicate 2	1.06	23.09	0.046
Technical replicate 3	2.21	22.89	0.096
Technical replicate 4	2.25	23.4	0.096
Technical replicate 5	1.32	23.08	0.057
**Average**	1.62	23.01	0.07
**Standard deviation**	0.57	0.29	0.024
