# Supplementary material for: A comprehensive collection of experimentally validated primers for Polymerase Chain Reaction quantitation of murine transcript abundance
Source: BMC Genomics. 2008 Dec 24;9:633. doi: 10.1186/1471-2164-9-633 (PMC2631021; doi:10.1186/1471-2164-9-633)
Supplement: Additional file 9 — Analysis of individual primer pairs from technical replicate experiments. [file 1471-2164-9-633-S9.pdf]

Well	Ct: technical replicate 1	Ct: technical replicate 2	Ct: technical replicate 3	Ct: technical replicate 4	Ct: technical replicate 5	Standard deviation	Average Ct	Coefficient of variation
A1	26.15	26.06	25.37	26.44	24.58	0.75	25.72	0.029
A2	22.2	23.65	22	23.09	23.17	0.7	22.82	0.03
A3	22.75	22.61	22.27	22.47	22.43	0.18	22.51	0.008
A4	22.11	22.93	20.92	22.79	23.74	1.05	22.5	0.047
A5	22.17	22.61	21.43	22.38	22.13	0.44	22.14	0.02
A6	21.89	22.02	21.11	22.1	22.62	0.54	21.95	0.025
A7	21.71	22.24	22.58	21.99	21.79	0.35	22.06	0.016
A8	21.67	22.23	20.29	23.22	22.14	1.07	21.91	0.049
A9	32.14	30.37	33.21	32.89	33.13	1.18	32.35	0.037
A10	22.18	22.39	21.87	22.27	22.32	0.2	22.21	0.009
A11	22.42	23.06	22.6	23.24	23.06	0.35	22.88	0.015
A12	22.35	22.51	22.69	22.92	23.4	0.41	22.77	0.018
B1	22.18	23.07	22.3	22.67	22.6	0.35	22.56	0.015
B2	22.9	23.38	22.67	23.23	23.17	0.28	23.07	0.012
B3	22.5	22.92	23.53	23.2	22.46	0.46	22.92	0.02
B4	22.37	22.38	21.75	22.19	22.18	0.26	22.17	0.011
B5	22.02	22.47	21.48	22.03	22.19	0.36	22.04	0.016
B6	21.79	22.46	21.37	22.45	22.04	0.46	22.02	0.021
B7	20.67	21.01	21.43	22.12	21.79	0.58	21.4	0.027
B8	22.02	22.69	22.25	22.52	22.47	0.26	22.39	0.012
B9	22.03	22.41	21.79	22.24	22.19	0.23	22.13	0.011
B10	22.1	22.58	21.29	22.22	22.32	0.49	22.1	0.022
B11	22.24	23.07	22.37	22.17	24.56	1	22.88	0.044
B12	22.44	22.86	22.35	23.02	22.47	0.29	22.63	0.013
C1	25.49	26.04	27.18	26.25	25.65	0.66	26.12	0.025
C2	23.18	23.68	23.3	23.49	23.87	0.28	23.5	0.012
C3	22.3	22.86	22.14	22.25	22.29	0.28	22.37	0.013
C4	22.26	22.88	22.26	Undetec.	22.7	0.31	22.52	0.014
C5	22.18	22.72	22.19	22.77	22.57	0.28	22.49	0.013
C6	22.09	22.48	21.93	21.96	23.33	0.59	22.36	0.026
C7	22.59	23.1	22.48	23.62	22.12	0.58	22.78	0.026
C8	22.12	22.62	22.02	21.66	22.55	0.4	22.19	0.018

C9	22.05	22.28	Undetec.	22.69	21.63	0.44	22.162	0.02
C10	23.39	23.78	23.05	23.49	24.42	0.51	23.63	0.022
C11	22.43	23.07	22.27	23.16	22.53	0.4	22.69	0.018
C12	22.88	23.21	22.93	23.14	23.04	0.14	23.04	0.006
D1	23.6	24.2	24.33	24.22	24.2	0.29	24.11	0.012
D2	23.17	23.58	23.18	23.61	23.52	0.22	23.41	0.009
D3	23.22	23.76	23.2	23.9	23.87	0.35	23.59	0.015
D4	23.04	23.52	23.13	23.28	23.16	0.18	23.23	0.008
D5	21.31	21.94	21.27	22.03	21.66	0.35	21.64	0.016
D6	22.28	23.01	22.35	23.01	23.57	0.53	22.84	0.023
D7	23.65	24.06	23.76	24.66	23.42	0.48	23.91	0.02
D8	22.25	23.03	22.52	22.79	23.07	0.35	22.73	0.015
D9	23.4	24.01	24.26	24.18	24.07	0.34	23.98	0.014
D10	22.46	22.72	22.24	23.39	22.92	0.44	22.75	0.019
D11	22.05	22.96	22.33	22.32	22.15	0.35	22.36	0.016
D12	21.23	21.26	20.34	21.02	21.09	0.37	20.99	0.018
E1	22.46	23.12	22.93	22.83	23.33	0.33	22.93	0.014
E2	22.69	22.84	22.64	23.37	22.6	0.32	22.83	0.014
E3	23.25	23.57	23.62	23.7	24.03	0.28	23.63	0.012
E4	22.78	23.39	25.05	23.09	23.01	0.91	23.46	0.039
E5	22.94	23.67	23.96	34.13	Undetec.	5.32	26.17	0.203
E6	22.92	23.31	22.64	22.27	23.17	0.42	22.86	0.018
E7	22.25	23.08	22.48	23.79	23.1	0.6	22.94	0.026
E8	21.49	22.2	21.8	21.73	22.13	0.29	21.87	0.013
E9	22.22	23.05	22.32	23.6	23.15	0.58	22.87	0.026
E10	22.29	22.92	22.28	22.2	22.45	0.29	22.43	0.013
E11	22.13	22.88	22.37	22.91	23.41	0.5	22.74	0.022
E12	23.14	23.68	23.88	24.32	23.25	0.48	23.65	0.02
F1	23.66	23.71	23.16	24.14	23.38	0.37	23.61	0.016
F2	23.24	23.6	23.38	23.45	23.74	0.19	23.48	0.008
F3	22.36	22.89	22.16	23.02	22.92	0.38	22.67	0.017
F4	22.36	22.72	22.92	22.91	23.21	0.31	22.82	0.014
F5	22.46	22.64	22.02	22.66	22.01	0.32	22.36	0.014

F6	22.46	22.94	22.36	23.21	22.82	0.35	22.76	0.015
F7	22.22	22.87	22.25	22.16	22.56	0.3	22.41	0.013
F8	22.72	22.98	22.35	23.21	23.16	0.35	22.88	0.015
F9	22.07	22.61	22.13	23.23	22.36	0.47	22.48	0.021
F10	21.51	22	22.87	23.53	22.56	0.78	22.49	0.035
F11	21.51	22.06	21.12	21.34	22.01	0.41	21.61	0.019
F12	22.39	23.05	22.02	22.04	22.9	0.48	22.48	0.021
G1	21.52	22.17	22.19	23.04	22.52	0.55	22.29	0.025
G2	24.09	24.24	23.72	24.17	24.04	0.2	24.05	0.008
G3	22.38	23.11	22.67	23.02	22.9	0.29	22.82	0.013
G4	23.16	23.05	22.44	22.78	22.84	0.28	22.85	0.012
G5	22.95	23.2	22.81	22.82	23.63	0.34	23.08	0.015
G6	22.64	22.85	22.32	22.79	22.26	0.27	22.57	0.012
G7	23	23.21	22.14	22.54	22.35	0.45	22.65	0.02
G8	22.42	23.19	22.76	24.12	23.23	0.64	23.14	0.028
G9	22.21	23.46	23.17	23.96	23.41	0.64	23.24	0.028
G10	22.15	22.95	22.24	22.5	22.79	0.34	22.53	0.016
G11	23.3	23.82	23.28	23.43	25.18	0.8	23.8	0.034
G12	22.2	22.72	22.69	31.32	23.12	3.88	24.41	0.159
H1	24.32	24.81	27.19	24.62	25.23	1.14	25.23	0.045
H2	22.27	23.01	23.05	24.02	23.08	0.62	23.09	0.027
H3	22.32	22.77	22.43	22.29	23.2	0.38	22.6	0.017
H4	22.92	23.3	22.99	23.64	22.83	0.33	23.14	0.014
H5	22.17	22.73	22.58	22.92	22.9	0.31	22.66	0.013
H6	22.69	23.19	23.02	23.34	23.21	0.25	23.09	0.011
H7	21.37	22.01	38.08	22.45	22.42	7.18	25.27	0.284
H8	22.49	22.53	Undetec.	22.28	22.66	0.16	22.49	0.007
H9	22.84	23.19	23.39	34.53	24.45	4.98	25.68	0.194
H10	22.16	22.65	22.03	22.59	22.55	0.28	22.4	0.012
H11	22.69	23.2	22.3	22.94	23.22	0.38	22.87	0.017
H12	21.96	22.58	22.27	22.56	22.8	0.32	22.43	0.014
**Average**						0.64	23.02	0.027
**Standard deviation**						1.04	1.34	0.04
