# Supplementary material for: A comprehensive collection of experimentally validated primers for Polymerase Chain Reaction quantitation of murine transcript abundance
Source: BMC Genomics. 2008 Dec 24;9:633. doi: 10.1186/1471-2164-9-633 (PMC2631021; doi:10.1186/1471-2164-9-633)
Supplement: Additional file 11 — Comparison of pipetting variation between manual and robotic liquid transfer. [file 1471-2164-9-633-S11.pdf]

**Pipetting conditions**	**Average value**	**Standard deviation**	**Coefficient of variation**	**Number of samples**
Manual transfer	5.56	0.11	0.019	12
Robotic transfer	5.29	0.15	0.028	96
