# Supplementary material for: A comprehensive collection of experimentally validated primers for Polymerase Chain Reaction quantitation of murine transcript abundance
Source: BMC Genomics. 2008 Dec 24;9:633. doi: 10.1186/1471-2164-9-633 (PMC2631021; doi:10.1186/1471-2164-9-633)
Supplement: Additional file 12 — Amplicons used for SYBR Green I sequence specificity experiments. [file 1471-2164-9-633-S12.pdf]

Additional file 12a. Amplicons of increasing length.

PrimerBank ID	AT%	Length (bp)
29179426a1	50	100
16945964a1	50	200
6754800a1	50	300
26339558a1	50	392
22128741a1	50	488
33238936a1	50	620
22129565a1	50	653
10567793a1	51	746

Additional file 12b. Amplicons of increasing AT%.

PrimerBank ID	AT%	Length (bp)
31982602a1	35	100
33859690a1	40	100
13386096a1	45	100
29179426a1	50	100
25072201a1	55	100
6679032a1	60	100
29789229a1	64	100
