# Supplementary material for: A comprehensive collection of experimentally validated primers for Polymerase Chain Reaction quantitation of murine transcript abundance
Source: BMC Genomics. 2008 Dec 24;9:633. doi: 10.1186/1471-2164-9-633 (PMC2631021; doi:10.1186/1471-2164-9-633)
Supplement: Additional file 14 — Amplification efficiency estimation from single reaction kinetics data. [file 1471-2164-9-633-S14.pdf]

Sample	Slope	Efficiency (%)	Ct	Cycle range used
33859690a1, 10 fg	0.84+/-0.02	84	20.05	19-21
26339558a1, 1 pg	0.79	79	25.58	25-26
16945964a1, 10 fg	0.93+/-0.01	93	21.11	20-22
22128741a1, 1 pg	0.81+/-0.01	81	25.78	25-27
25072201a1, 10 fg	0.91+/-0.02	91	19.69	19-21
6679032a1, 10 fg	0.94+/-0.01	94	20.01	19-21
13386096a1, 10 fg	0.96+/-0.01	96	20.29	19-21
29789229a1, 10 fg	0.93+/-0.02	93	19.02	18-20
22129565a1, 100 fg	0.91+/-0.02	91	23.25	22-24
6754800a1, 1 pg	0.83+/-0.01	83	22.93	22-24
31982602a1, 100 fg	0.86+/-0.01	86	21.12	20-22
33238936a1, 100 fg	0.89	89	22.57	22-23
29179426a1, 100 fg	0.9	90	19.54	19-20
