# Supplementary material for: A comprehensive collection of experimentally validated primers for Polymerase Chain Reaction quantitation of murine transcript abundance
Source: BMC Genomics. 2008 Dec 24;9:633. doi: 10.1186/1471-2164-9-633 (PMC2631021; doi:10.1186/1471-2164-9-633)
Supplement: Additional file 15 — Amplification efficiency estimation using analytical and standard curve methods. [file 1471-2164-9-633-S15.pdf]

Standard curve method			
**Sample**	**Efficiency (%)**	**P value**	**95% Confidence intervals (slope)**
33859690a1	133	0.0002	-1.67 to -1.00
26339558a1	73	0.0015	-0.99 to -0.47
16945964a1	99	<0.0001	-1.18 to -0.80
22128741a1	94	0.0019	-1.30 to -0.58
25072201a1	103	<0.0001	-1.17 to -0.90
6679032a1	103	<0.0001	-1.11 to -0.94
13386096a1	103	<0.0001	-1.12 to -0.94
29789229a1	106	<0.0001	-1.12 to -1.00
22129565a1	98	<0.0001	-1.13 to -0.82
6754800a1	92	0.0056	-1.43 to -0.41
31982602a1	77	<0.0001	-0.90 to -0.63
33238936a1	88	<0.0001	-1.00 to -0.76
29179426a1	106	0.0001	-1.26 to -0.86
**Average efficiency**	98		
**Standard deviation**	15		
**Range of efficiency**	73 to 133		
**Variance**	223		

Analytical method			
**Sample**	**Efficiency (%)**	**P value**	**95% Confidence intervals (slope)**
33859690a1	84	0.0185	0.53 to 1.15
26339558a1	78	<0.0001	0.76 to 0.81
16945964a1	93	0.0088	0.77 to 1.10
22128741a1	81	0.0067	0.70 to 0.92
25072201a1	91	0.0134	0.67 to 1.15
6679032a1	94	0.0104	0.75 to 1.14
13386096a1	93	0.0004	0.85 to 1.01
29789229a1	93	0.0139	0.67 to 1.19
22129565a1	89	0.0004	0.81 to 0.96
6754800a1	83	0.007	0.72 to 0.95
31982602a1	86	0.0105	0.86+/-0.01
33238936a1	88	0.0018	0.88
29179426a1	86	0.0147	0.61 to 1.11
**Average efficiency**	88		
**Standard deviation**	5		
**Range of efficiency**	78 to 94		
**Variance**	26		
