# Supplementary material for: A comprehensive collection of experimentally validated primers for Polymerase Chain Reaction quantitation of murine transcript abundance
Source: BMC Genomics. 2008 Dec 24;9:633. doi: 10.1186/1471-2164-9-633 (PMC2631021; doi:10.1186/1471-2164-9-633)
Supplement: Additional file 17 — PrimerBank primer pair groups used for one-way ANOVA analysis. [file 1471-2164-9-633-S17.pdf]

**Group**	**Average efficiency**	**Standard deviation**	**Coefficient of variation**
33859690a1	93	13	0.143
26339558a1	82	14	0.172
16945964a1	116	55	0.479
22128741a1	79	1	0.014
25072201a1	131	94	0.714
6679032a1	95	16	0.166
13386096a1	94	17	0.183
29789229a1	97	16	0.166
22129565a1	94	39	0.416
6754800a1	108	65	0.605
31982602a1	99	40	0.404
33238936a1	88	11	0.131
29179426a1	89	5	0.054
